# Effect of Exercise on Inflammation in Hemodialysis Patients: A Systematic Review

**DOI:** 10.3390/jpm12071188

**Published:** 2022-07-21

**Authors:** Erika Meléndez Oliva, Jorge H. Villafañe, Jose Luis Alonso Pérez, Alexandra Alonso Sal, Guillermo Molinero Carlier, Andrés Quevedo García, Silvia Turroni, Oliver Martínez-Pozas, Norberto Valcárcel Izquierdo, Eleuterio A. Sánchez Romero

**Affiliations:** 1 Musculoskeletal Pain and Motor Control Research Group, Faculty of Sport Sciences, Universidad Europea de Madrid, 28670 Madrid, Spain; erikamelendezoliva@gmail.com (E.M.O.); joseluis.alonso@univer-sidadeuropea.es (J.L.A.P.); alexandraalonsosal@hotmail.com (A.A.S.); gmolineroc@hotmail.com (G.M.C.); queveglez@hotmail.com (A.Q.G.); oliver.martp@gmail.com (O.M.-P.); 2 Musculoskeletal Pain and Motor Control Research Group, Faculty of Health Sciences, Universidad Europea de Canarias, Tenerife, 38300 Canary Islands, Spain; 3 IRCCS Fondazione Don Carlo Gnocchi, Piazzale Morandi 6, 20148 Milan, Italy; 4 Department of Physiotherapy, Faculty of Sport Sciences, Universidad Europea de Madrid, Villaviciosa de Odón, 28670 Madrid, Spain; 5 Department of Physiotherapy, Faculty of Health Sciences, Universidad Europea de Canarias, Tenerife, 38300 Canary Islands, Spain; 6 Onelifecenter, Multidisciplinary Pain Treatment Center, 28925 Madrid, Spain; 7 Department of Pharmacy and Biotechnology, University of Bologna, Via Belmeloro 6, 40126 Bologna, Italy; silvia.turroni@unibo.it; 8 Higher Institute of Medical Sciences of Havana Victoria de Girón, University of Medical Sciences of Havana, Havana 11600, Cuba

**Keywords:** chronic kidney disease, hemodialysis, inflammation, exercise

## Abstract

Background: In recent years, physical exercise has been investigated for its potential as a therapeutic tool in patients with end-stage renal disease (ESRD) undergoing hemodialysis maintenance treatment (HD). It has been shown that regular practice of moderate-intensity exercise can improve certain aspects of immune function and exert anti-inflammatory effects, having been associated with low levels of pro-inflammatory cytokines and high levels of anti-inflammatory cytokines. Purpose: The aim of this review is to examine the studies carried out in this population that analyzed the effect of intradialytic exercise on the inflammatory state and evaluate which exercise modality is most effective. Methods: The search was carried out in the MEDLINE, CINAHL Web of Science and Cochrane Central Register of Controlled Trials databases from inception to June 2022. The PEDro scale was used to assess methodological quality, and the Cochrane Risk of Bias Tool and MINORS were used to evaluate the risk of bias. The quality of evidence was assessed with GRADE scale. The outcome measures were systemic inflammation biomarkers. Results: Mixed results were found in terms of improving inflammation biomarkers, such as CRP, IL-6 or TNFα, after exercise. Aerobic exercise seems to improve systemic inflammation when performed at medium intensity while resistance training produced better outcomes when performed at high intensity. However, some studies reported no differences after exercise and these results should be taken with caution. Conclusions: The low quality of the evidence suggests that aerobic and resistance exercise during HD treatment improves systemic inflammation biomarkers in patients with ESRD. In any case, interventions that increase physical activity in patients with ESRD are of vital importance as sedentary behaviors are associated with mortality. More studies are needed to affirm solid conclusions and to make intervention parameters, such as modality, dose, intensity or duration, sufficiently clear.

## 1. Introduction

Chronic kidney disease (CKD) is related to severe impairments of innate and adaptive immunity, resulting in a complex state of immune dysfunction in which signs of immune depression and activation paradoxically coexist [1,2]. Although functional immune cell imbalances susceptible CKD patients to infectious complications, persistent immune cell activation promotes a chronic inflammatory state that is related to an elevated risk of cardiovascular disease (CVD). Given that CVD and infections are the leading causes of morbidity and mortality in patients with CKD [3,4]. the immune dysfunction that accompanies CKD constitutes a major target for improving the outcomes of therapeutic interventions.

In recent years, the potential of physical exercise as a therapeutic tool in patients with end-stage renal disease (ESRD) undergoing hemodialysis (HD) maintenance treatment has been investigated [5]. Regular practice of moderate-intensity exercise has been shown to improve certain aspects of immune function and exert anti-inflammatory effects [6,7], having been associated with low levels of pro-inflammatory cytokines and high levels of anti-inflammatory cytokines [8]. These effects contribute, at least in part, to reducing the risk of infections and CVD [6,7]

The persistent inflammation and immune dysregulation present in CKD patients on HD can result in a loss of skeletal muscle mass, muscle strength and functional capacity. Exercise produces mechanical stress on the muscle fiber, which can alter the inflammatory response that affects structural and functional adaptation, remodeling and repairing processes in the skeletal muscles [9,10] On the other hand, physical activity can induce the production of anti-inflammatory cytokines and a selective decrease in circulating CD16 + monocytes through a transient increase in endogenous glucocorticoids [11]. In addition, exercise has been shown to decrease inflammation-dependent Toll-like receptors (TLRs), reducing cellular expression of TLR4 and consequently mitigating the inflammatory response induced by lipopolysaccharides [12].

Furthermore, it is very common for ESRD patients to have high levels of physical inactivity in their daily life, exacerbating chronic inflammation and loss of muscle mass and strength, which, associated with high cardiovascular morbidity, can create a vicious cycle in these patients [13].

In an attempt to clarify the state of the art on this topic, here, we conducted a systematic review of the literature to investigate the most effective exercise modality for reducing the inflammatory state of ESRD patients on HD, and, consequently, their cardiovascular morbidity and mortality while improving physical function and health-related quality of life.

## 2. Materials and Methods

This is a systematic review of studies researching the effect of exercise on inflammatory status in patients with ESRD on HD. PRISMA [14]. guidelines were followed during the design, search and reporting stages of this review. The protocol for this systematic review was registered on PROSPERO (CRD42021288811).

### 2.1. Search Strategy

A literature search was conducted to identify all available studies on the effect of exercise on inflammatory status in ESRD patients on HD, with no publication date limit until September 2021 and no language limit, in the PubMed, CINAHL, Web of Science and Cochrane Central Register of Controlled Trials databases. The PubMed search strategy was (“exercise”[MeSH Terms] OR “exercise”[All Fields] OR “exercises”[All Fields] OR “exercise therapy”[MeSH Terms] OR (“exercise”[All Fields] AND “therapy” [All Fields]) OR “exercise therapy”[All Fields] OR “exercise s”[All Fields] OR “exercised”[All Fields] OR “exerciser”[All Fields] OR “exercisers”[All Fields] OR “exercising”[All Fields]) AND (“inflammation”[MeSH Terms] OR “inflammation”[All Fields] OR “inflammations”[All Fields] OR “inflammation s”[All Fields]) AND (“haemodialysis”[All Fields] OR “renal dialysis”[MeSH Terms] OR (“renal”[All Fields] AND “dialysis”[All Fields]) OR “renal dialysis”[All Fields] OR “hemodialysis”[All Fields]). For other databases, the string was adjusted if necessary. We searched for additional records through other sources to supplement the database findings; for example, we hand-searched the reference lists of the relevant literature reviews and the indexes of peer-reviewed journals.

Five independent researchers (E.M.O., A.A.S., O.M.P., G.M.C. and A.Q.G.) conducted the searches and evaluated all the articles found by title and abstract and, subsequently, the full-text publications to determine their eligibility. This procedure was performed by each researcher involved in this part of the study (E.M.O., A.A.S., O.M.P., G.M.C. and A.Q.G.) and according to the inclusion and exclusion criteria of the research. A sixth author (E.A.S.R) resolved any discrepancies. The reference list of each article was screened to find any additional original articles.

### 2.2. Study Selection

#### 2.2.1. Type of Studies

The types of studies included randomized controlled trials, non-randomized controlled trials, uncontrolled trials, non-randomized clinical trials, randomized crossover trials, and non-randomized crossover trials in ESRD patients on HD who underwent exercise intervention whose effect on inflammatory levels was evaluated, without restrictions regarding the date of publication. We excluded all repeated articles, case reports, letters to the editor, pilot studies, editorials, technical notes and review articles from the analysis. Articles written in any language were included.

#### 2.2.2. Type of Participants

Participants in selected studies had to be adults (aged 18 years or older) with a diagnosis of ESRD and undergoing HD for at least 3 months.

#### 2.2.3. Data Extraction

Five authors (E.M.O., A.A.S., O.M.P., G.M.C. and A.Q.G.) conducted the data extraction independently. A sixth author (E.A.S.R.) resolved any discrepancies. Reviewers were not blind to information regarding the authors, journal or outcomes for each article reviewed. A standardized form was used to extract data concerning study design, number and mean age of participants, the proportion of males/females, year and country of publication, setting, association of exercise with inflammatory levels, follow-up timing, clinical outcome measures and reported findings. Following the guidelines of the Cochrane Handbook for Systematic Reviews of Interventions—Version 5.1.0, the form was developed. It was pilot tested for reliability based on a representative sample of the studies to be screened.

#### 2.2.4. Quality Assessment

The methodological quality of the clinical trials was assessed using the PEDro scale, reinforced by the use of the Cochrane Risk of Bias Tool for the evaluation of the risk of bias, in order to provide more detail in the evaluation.

The methodological index for non-randomized studies (MINORS) [15] was used to assess the methodological quality and risk of bias of the included studies. This scoring system includes eight items for non-randomized studies and four additional items for comparative studies. Each item is scored between 0 and 2, and the maximum attainable score is 16 and 24 for non-randomized and comparative studies, respectively. Five authors independently answered questions with 0 (not reported), 1 (reported but inadequate) or 2 (reported and adequate); any disagreements were resolved through discussion and, if no consensus was reached, another review author was consulted and a decision made.

#### 2.2.5. Certainty of Evidence

The certainty of the evidence analysis was established by different levels of evidence according to the Grading of Recommendations, Assessment, Development and Evaluation (GRADE) framework, which is based on five domains: study design, imprecision, indirectness, inconsistency and publication bias [16]. The evidence was classified into the following four levels: high quality (all five domains are satisfied), moderate quality (one of the five domains are not satisfied), low quality (two of the five domains are not satisfied) or very low quality (three of five domains are not satisfied) [17].

For the risk of bias domain, recommendations were downgraded one level if there was unclear or high risk of bias and severe limitations in the estimation effect. For consistency, recommendations were downgraded when point estimates varied widely among studies, confidence intervals overlapped or when the I^2^ test was substantial (>50%). For the indirectness domain, when serious differences in interventions, populations or outcomes were found, they were downgraded by one level. For the imprecision domain, if there were fewer than 300 participants for key outcomes, it was downgraded one level. Finally, if a strong influence of publication bias was detected, one level was downgraded [18].

## 3. Results

### 3.1. Study Selection

**Figure 1:**
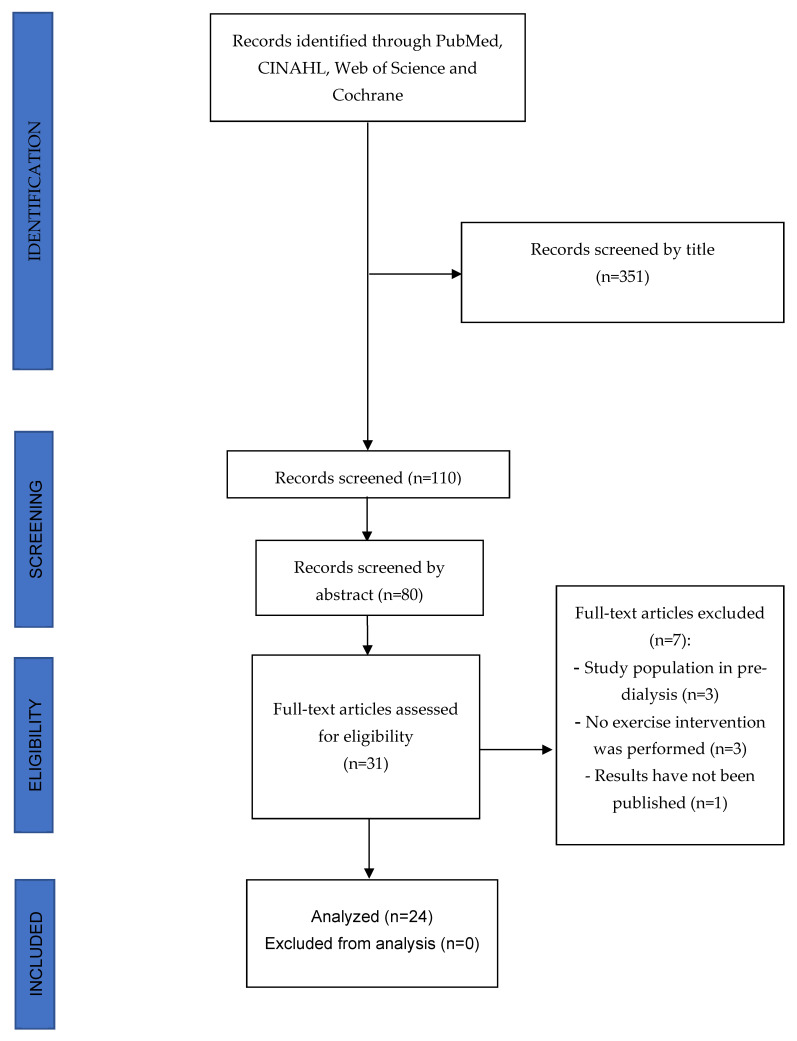
PRISMA Flow diagram.

A total of 351 studies were detected and analyzed by performing the proposed searches in the detailed databases. After removing duplicates and analyzing the titles and abstracts of the remaining articles, 31 full-text articles were evaluated for possible inclusion in the present study. Finally, 9 of these manuscripts were excluded because they were not carried out in patients with ESRD on HD because they did not perform an exercise program as an intervention or because their results had not yet been published. Therefore, 24 studies were ultimately selected for this review (see Figure 1 for the PRISMA flow diagram).

The 24 studies included were conducted in Iran [19], Brazil [20,21,22,23,24,25,26,27,28,29], Poland [30], the United Kingdom [31,32,33,34,35,36], Taiwan [37], China [38], Indonesia [39], Spain [40,41] and Greece [42], and published from 2010 to 2022.

### 3.2. Study Characteristics

The characteristics of the included studies are presented in Table 1. The 24 studies were clinical trials, of which thirteen were randomized controlled trials [19,25,26,27,28,29,35,36,37,38,39,41,42], four were uncontrolled trials [20,21,30,40], one was a non-randomized clinical trial [23], one was a non-randomized controlled trial [32], four were randomized crossover trials [22,24,31,33], and one was a non-randomized crossover trial [34].

### 3.3. Methodological Quality and Risk of Bias of the Included Studies

The methodological quality of the studies was evaluated with the PEDro scale, and scores are shown in Table 2. In total, seventeen studies were evaluated as being of excellent quality [19,22,24,25,26,27,28,31,33,34,35,36,37,38,39,41,42], two studies were of good quality [23,29] and five studies were of acceptable quality [20,21,30,32,40].

The risk of bias of the randomized controlled trials was evaluated with the Cochrane Risk of Bias Tool, and scores are shown in Table 3. In total, seventeen studies were evaluated. Five studies were at low risk of bias [26,28,31,37,39], five studies had an unclear risk [25,35,36,41,42] and seven studies were at high risk of bias [19,22,24,27,29,33,38].

For non-randomized controlled trials, the MINORS scale was used, and scores are shown in Table 4. In total, seven studies were evaluated. Six studies scored above 12/24 points and showed good quality [21,23,30,32,34,40], and only one study scored below 12/24 points with fair quality [20].

### 3.4. Quality of Evidence

Quality of evidence of AE and RT was assessed with the Grading of Recommendations, Assessment, Development and Evaluation (GRADE) framework, and results are shown in Table 5.

The low quality of the evidence supports the use of AE and RT exercises in patients with ESRD to improve biomarkers of systemic inflammation.

### 3.5. Data from Studies

The most relevant results obtained in the studies included in the review are mentioned below:

#### 3.5.1. Effect of Exercise on Main Inflammatory Biomarkers

##### C-Reactive Protein

Fourteen studies analyzed the effects of exercise on CRP levels with different results. One study did not find differences in CRP levels after AE [30] and two studies did not find differences after RT [23,40]. Additionally, one study found no differences when AE were compared to RT [39] or when compared to usual care [24,27,32,35]. On the other hand, two studies found decreased levels of CRP after AE compared to usual care [33,37], one study found improvements favoring AE when compared to RT [19] and two studies found improvements after RT exercise [20,21]. Finally, when AE were combined with RT, one study reported decreased levels after intervention [41].

##### IL-6

Twelve studies analyzed the effects of exercise on IL-6 levels with different results. One study did not find differences in IL-6 after AE [30] and three studies did not find differences after RT [20,21,28]. Additionally, no changes were found when comparing AE and RT in IL-6 levels [26] or when compared to usual care [31,32,35,36]. On the other hand, two studies found decreased levels of IL-6 after AE compared to usual care [25,37]. Lastly, when AE were combined with RT, IL-6 levels were decreased after intervention [41].

##### TNFα

Twelve studies analyzed the effects of exercise on TNFα levels with different results. Three studies found no changes after AE [30] and three studies found no differences after RT [20,21,28]. No significant changes were reported when AE was compared to usual care in terms of TNFα improvements [31,32,35,36]. However, two studies found reduced TNFα levels after AE compared to usual care [25,33] and two studies reported decreased TNFα levels after RT compared to usual care [29,38].

#### 3.5.2. Effect of Exercise on Other Inflammatory Biomarkers

Other biomarkers less studied were IL-10, IL-1β, IL-17a, IL-8, IL-1ra, IFN-γ, ROS and SOD.

Five studies analyzed the effects of exercise on IL-10, two of them reporting increases after AE [22,25] and another one reporting no changes after RT [28]. Likewise, AE compared to the control did not improve IL-10 levels [35,36]. 

Two studies evaluated IL-1β levels after AE, with one study reporting reduced levels of IL-1β after AE [25] and another one not finding any differences [30].

One study found decreased levels of IL-17a during HD, but it increases after HD in patients who did not performed exercise [22]. However, another study found no significant differences when AE was compared to usual care improving IL-17a [36].

Other studies analyzed exercise on biomarkers IL-8 and IFN-γ, reporting decreased levels of IL-8 after AE [25], as well as decreased levels of IFN-γ after AE [22].

Two studies evaluated IL-1ra levels after AE, reporting no changes after AE [30,31].

Finally, one study found increased ROS levels after AE and at 60 min (but no later), suggesting a transient pro-inflammatory event [34]. On the other hand, RT reduced antioxidant enzymes after exercise, but increased on the day after exercise [23].

## 4. Discussion

The main objective of this systematic review was to synthesize the evidence of the effects of exercise on inflammatory mediators in patients with CKD at ESRD and HD. The different exercise approaches analyzed were the following: aerobic exercise and resistance training. The main inflammatory mediators analyzed were CPR, TNFα and IL-6, which are highly predictive of mortality in these patients, therefore, they were our focus on the present review [43].

### 4.1. Aerobic Exercise

Thirteen studies analyzed the effects of AE in CKD patients undergoing HD, with a total of 351 patients analyzed [22,24,25,27,30,31,32,33,34,35,36,37,42]. Most studies had excellent methodological quality, although the risk of bias ranged from high to low, with many studies scoring a high risk of bias and only two studies presenting a low risk of bias [31,37]. All of them used the cycloergometer as aerobic training with a total exercise duration from 30 to 60 min. Most studies performed at medium intensity (12–15 RPE).

Regarding the effectiveness of AE on inflammatory mediators, mixed evidence is found. With respect to IL-6, Liao et al., and Gonçalves da Cruz et al., found decreased IL-6 after 36 sessions of AE at medium intensity [25,37]. In contrast, Dungey et al., found no differences in inflammatory mediators after fewer sessions of AE [31]. However, other studies with longer training periods also found no differences [30,32,35,36]. 

The same scenario occurs in relation to CPR and TNFα, with studies reporting decreased levels after AE [37,42], and others without differences [24,27,35]. 

Other less investigated mediators such as IL-17a, IFNγ or IL-1ra seem to be unaffected by AE [22,31,36], while others such as IL-10 seem to increase after AE [22,25]. Finally, one study found that acute AE increased ROS (Reactive Oxygen Species), suggesting a proinflammatory response after acute exercise [34]. However, these results should be taken with caution due to the low sample size and small number of studies.

There is no clear evidence that AE reduce inflammatory mediators associated with CKD, such as IL-6, TNFα or CRP. Regarding the effectiveness of AE on other inflammatory conditions, such as COVID-19, a recent review found that aerobic training at medium intensity, from approximately 20 to 60 min and performed three times per week, improves inflammatory mediators [44]. We suggest that exercise intensity could be a parameter that could explain these differences found in patients with CKD and ESRD, as high intensity training can increase IL-6 levels compared to medium intensity training [45]. The implementation of future long-term and medium-intensity based training programs could shed light on whether AE can reduce inflammatory mediators.

### 4.2. Resistance Exercise

Seven studies analyzed the effects of RT in CKD patients undergoing HD, with a total of 316 patients analyzed [20,21,23,28,29,38,40]. Methodological quality was from acceptable to excellent, and the risk of bias was from low to high, with only one study with a low risk of bias [28]. Most studies used lower limb strengthening programs, although some added upper limbs as well [29,40]. Four studies did not specify exercise intensity, while others worked from 60 to 70% 1RM or high intensity training (>15 RPE).

Regarding the effectiveness of RT on the aforementioned inflammatory mediators, again, mixed results are found.

With respect to IL-6, none of the analyzed studies reported changes in IL-6 when RT was performed, all including long-term (36–72 weeks) moderate–high intensity training [20,21,28,38]. In comparison, healthy adults who performed RT had significant decreases in IL-6 after RT [46]. In addition, Gadelha et al., found that CKD patients performing RT showed a decrease in IL-6 levels after 24 weeks of moderate-intensity training [47]. Differences with our results might be associated with training intensity as well as training programs because Gadelha et al., performed whole-body exercises.

With reference to CRP, three studies found decreases after RT and two did not. Moraes et al., found decreases in CRP after RT at 70% 1RM (20), while Dong et al., found the same results after high intensity RT (>15 RPE) [38]. However, among studies that did not find differences, two of them did not report exercise intensity and the other one performed at medium intensity [23]. This is in line with a recent published meta-analysis that found that RT had larger effect sizes improving CRP when it was performed at vigorous intensity [46].

The same results are found when evaluating TNFα. Three studies found no differences while another two studies found decreases after RT [20,21,28,38]. The studies that reported no differences were performed at high intensity and for long periods of time, but those that found differences also did so, suggesting that intensity may not be a key factor in decreasing TNFα. Studies suggest that specific populations (such as heart failure) may benefit more from RT than others [46].

Other mediators such as VCAM-1, ICAM-1 and oxidative stress mediators were reduced after RT [21,23]. Two studies found no differences in IL-10 after RT, while one study found increases afterwards, although results should be taken with caution due to low sample sizes [28,29,38]. 

We found clear evidence of a reduction in CRP in patients with CKD who performed high-intensity RT. However, we found no changes in IL-6 levels after long periods of RT and mixed evidence in terms of TNFα. Although high intensity training suggests better results for decreasing inflammatory mediators, future studies are required.

### 4.3. Compared and Combined Interventions

Two studies compared AE vs. RT in CKD patients undergoing HD, with a total of 58 patients analyzed [19,26]. All studies had excellent methodological quality and risk of bias ranging from low to high, with only one study presenting a low risk of bias [26].

Afsar et al., found that both interventions reduced CRP compared to the baseline, but with better results in favor of AE [19]. In terms of Il-6, Figueredo et al., reported slight improvements in IL-6 but without differences between groups [26].

Two studies combined AE with RT in CKD patients undergoing HD, with a total of 191 patients analyzed [39,41]. All studies had excellent methodological quality and risk of bias ranging from unclear to low, with only one study presenting a low risk of bias [41].

Regarding CRP, Suhardjono et al., found no differences when AE was combined with RT in terms of CRP decrease [39], while Meléndez et al., found decreases in CRP levels after intervention [41]. With respect to IL-6, combined exercise reduced IL-6 levels [41].

Strategies that combine AE with RT may exert better outcomes than both modalities alone. In line with these findings, Sadjapong et al., found that elderly patients with chronic inflammation who performed AE at medium intensity and RT at high intensity improved their levels of IL-6 and CRP more than a control group receiving usual care [47]. Our results show similar effects, with a greater improvement in IL-6 after AE and improvements in CRP after RT, so that combining both exercises could be more beneficial than performing them separately. However, the limited number of studies makes it difficult to draw strong conclusions. Future studies should evaluate the efficacy of a combined intervention in CKD patients with ESRD under HD.

### 4.4. Future Directions

Although more studies are still needed to analyze the effects of exercise on inflammatory levels in patients with ESRD on HD, it is well-demonstrated in this review that exercise has a beneficial effect by decreasing different inflammatory cytokines and improving oxidative stress in this population. However, it is not clear which is superior.

It seems that medium-intensity training produces better results in patients who perform AE and high intensity training in patients who perform RT, hence, adjusting the intensity of training is a key point for these patients. In addition, acute exercise can increase some proinflammatory mediators so longer periods of training may be beneficial in developing long-term anti-inflammation adaptation [7]. Combining both trainings might be a better strategy than performing them separately, but again, intensity must be regulated. Regular AE may promote an anti-inflammatory environment, decreasing proinflammatory mediators and RT, in addition to favoring the same environment and improving muscular mass and functional capacity [48].

Finally, CKD is a multifactorial disease and must be approached in a multidisciplinary way. Sarcopenia is commonly found in CKD patients, and it is associated with disease progression and mortality. Combining strategies such as exercise and nutritional advice are highly recommended. For example, patients with CKD who underwent RT and a nutritionist-supervised diet showed decreases in IL-6, emphasizing the importance of nutrition in these patients [49]. Adding nutritional behavioral changes (such as increasing protein intake) to exercise programs could help clinicians to slow down sarcopenia in CKD patients, although more studies are needed [50].

### 4.5. Limitations

Due to the great heterogeneity of the included studies, no meta-analysis of the results has been carried out, which should be considered as a limitation of the study. However, the systematic review carried out amply responds to the stated objectives.

Regardless of the exercise intervention, it should be noted that most of the included studies had a high risk of bias, with allocation concealment and blinding of participants/therapist being the lowest scoring items. In contrast, the methodological quality of included studies was good to excellent in most of them.

## 5. Conclusions

Some studies have shown the positive effect of exercise in reducing the inflammatory state of HD patients, achieving better benefits with long-duration programs with an intensity >15 on the scale of perceived exertion (RPE).

However, other studies were presented in which exercise had no effect on these parameters, which calls for caution in drawing strong conclusions to avoid overestimating its effect.

In any case, interventions that increase physical activity in patients with ESRD are of vital importance as sedentary behaviors are associated with mortality. The low quality of evidence supports the inclusion of exercise programs in patients with ESRD undergoing HD; although, more studies are needed to affirm solid conclusions and make intervention parameters such as modality, dose, intensity, or duration sufficiently clear.

## Figures and Tables

**Table 1:** The effect of exercise on inflammation in HD patients.

Author, Year	Study Design	Aim of the Study	Participants	Treatment	Outcome Measures	Reported Results
**Afsar et al. (2010)** [19]	Randomized controlled trial	To establish the effects of resistance and aerobic exercises on blood lipids and inflammation state in HD patients.	21 HD patients (males) were randomized into 3 groups: aerobic exercise (*n* = 7; mean age 50.7 ± 21.06 years), resistance training (*n* = 7; mean age 51 ± 16.4 years) and control group (*n* = 7; mean age 53 ± 19.4 years). Inclusion criteria: maintenance HD >3 months; age >20 years.	8 weeks intradialytic exercise program (3 times/week). Aerobic group: 10–30 min stationary cycling at an intensity of 12–16 at RPE (65–85% individual’s maximal capacity). Resistance group: ankle weights for knee extension, hip abduction and flexions at an intensity of 15–17 at RPE (60% of 3RM for 2 sets of 8 repetitions and was increased to 3 sets as tolerated).	-Inflammation measures: hs-CRP. -Other measures: BMI, Kt/V, creatinine, serum urea, albumin, hemoglobin and lipid levels (triglycerides, HDL-C, LDL-C and total cholesterol). -Exercise adherence: Not described.	Aerobic and resistance exercises were correlated with hs-CRP levels (*p* = 0.005 and *p* = 0.036) and serum creatinine (*p* < 0.0001 and *p* < 0.001) reduction so that aerobic exercise induced more reduction (*p* < 0.001 between groups). There were no changes in BMI, Kt/V values, serum urea, albumin, hemoglobin and lipid levels (*p* > 0.05).
**Moraes et al. (2012)** [20]	Uncontrolled trial	To analyze the effects of a resistance exercise (RE) program on biochemical parameters, inflammation markers and body composition in HD patients.	36 HD patients (61.1% men, mean age 46.7 ± 2.5 years) were studied. All patients exercised at the same time in a single group.	6-month RE program was performed to reduce inflammation and improve nutritional status in HD patients. Type of exercise and intensity are not specified.	-Biochemical parameters: albumin, globulin, creatinine, IL-6, TNF-α and CRP. -Anthropometric parameters: BMI, AMA, body fat and lean mass.	-Statistically significant improvements were observed in body composition (AMA, body fat and lean mass, *p* < 0.001), albumin (*p* < 0.001) and CRP levels (*p* < 0.001). -No significant changes were observed for BMI, creatinine, IL-6 and TNF-α.
**Golebiowski et al. (2012)** [30]	Uncontrolled trial	To assess the influence of cycling exercise during HD on patients’ physical capacity, muscle strength, quality of life and some laboratory parameters.	29 patients (15 men; mean age 64.2 ± 13.1 years; 4–192 months on HD maintenance) were recruited. Inclusion criteria: maintenance HD >3 months; age >18 years, and hemoglobin level >8 g/dL.	3-month intradialytic exercise program by cycle ergometer (3 times/week). Each time patients started exercising after the beginning of HD sessions and continued for 50 ± 19 min. The physical load was individually adapted to exercise tolerance (the energy expenditure during one session with exercise was diverse and amounted to 403 ± 219 kJ).	-Inflammatory parameters: hs-CRP, IL-6, IL-1β, IL-1ra and TNF. -Parameters of nutrition and lipid metabolism: BMI, albumin, HDL, LDL and TG. -Muscle strength of lower extremities: isokinetic knee extension and flexion peak torque. -Physical capacity: 6-minute walk test (6MWT). -Quality of life: SF-36 questionnaire. -Exercise adherence. Measures were taken at T_0_ and T_3._	The exercise program did not produce significant changes in nutrition and inflammation parameters (*p* > 0.05). In the 6-minute walk test, the increase in walk velocity was 4% (*p* < 0.01). At angular velocity (AV) of 60°/s, extension peak torque in the knee joint rose by 7% and at AV of 300°/s by 4% (*p* = 0.04). Flexion peak torque at AV of 180°/s increased by 13% (*p* = 0.0005). Exercise adherence: 72.4% (21 of 29 patients completed the exercise program).
**Moraes et al. (2014)** [21]	Uncontrolled trial	To assess the effects of 6 months of an intradialytic resistance exercise training program (RETP) on inflammation biomarkers, physical activity and protein-energy wasting (PEW) in patients undergoing HD.	52 HD patients started RETP, and 41 (78,4%) completed the program. A total of 37 patients (56.7% men, 45.9 ± 14.1 years, 23.5 ± 3.9 kg/m^2^) had their blood collected at the end of RETP for biomedical analysis. Inclusion criteria: age >18 years and patients on maintenance HD for at least 6 months. Patients who complied with <75% (<54 exercise sessions) of RETP were excluded.	Participants performed 6 months of intradialytic RETP. Exercise was performed during the first 2 h of HD, 3 times per week for 6 months (72 sessions). The exercises were performed in both limbs with elastic bands, and the intensity was based on the 1RM; the initial intensity was 60% of 1RM and according to the patients’ performance, the intensity reached 70% of 1RM. Patients rested 1 min between the 4 sets of 10 repetitions, and 3 min between the exercise categories.	-Plasma adhesion molecules levels: ICAM-1 and VCAM-1. -Inflammation biomarkers: IL-6, CRP and TNF-α. -Anthropometric: body weight (kg), height (m), WC, skinfold measurement (mm) (biceps, triceps, subscapular and suprailiac), muscle area and body fat. -Nutritional status: SGA (quantitative score system). -Physical capacity: muscle strength (using an isokinetic dynamometer). -PEW (simultaneously presence of BMI <23 kg/m^2^, serum albumin and reduced arm muscle area). -Adherence exercise.	-After 6 months of RETP, decreased ICAM-1 (*p* < 0.05), decreased VCAM-1 (*p* < 0.05) and decreased CRP levels (*p* < 0.001). -Body composition improved, albumin increased (*p* < 0.05) and the number of patients presenting PEW was decreased (*p* = 0.005). -IL-6 and TNF-α did not undergo significant changes. -Adherence exercise: 37 patients performed 75% of the sessions scheduled in the program (≥54 sessions). Only 2 subjects were excluded for not reaching this percentage.
**Peres et al. (2015)** [22]	Randomised controlled crossover trial	To evaluate the acute inflammatory response to intradialytic exercise in the peripheral blood of individuals with ESRD.	9 HD patients of 64.88 ± 1.98 years, of both genders (77.8% female).	Participants were randomly assigned to perform 2 HD sessions in different conditions with an interval of 1 week between each: -Aerobic intradialytic exercise sessions (EX), conducted by a physiotherapist and consisting of an HD session with aerobic exercise on a lower limb cycle ergometer. Exercise was performed after the first 2 h of HD, with a duration of 20 min to 6–7 in the Modified Borg Scale (MBS). -A control HD session (CON), when the subjects performed a conventional HD session lasting 4 h, without exercise.	-Peripheral blood collection was made at T_0_, during and immediately after HD to evaluate the cytokine profile: IL-6, IL-10, IL-17a, IFN-γ and TNF-α. -Physical capacity: 6MWT. -Anthropometric measures: body mass, height, abdominal circumference (AC) and BMI.	-IFN-γ decreased during HD when compared with the pre-moment in both sessions (*p* = 0.001), while an increase in post-HD was only found in the CON session (*p* = 0.001). -IL-17a was higher in the post when compared with during HD in both sessions (*p* = 0.004 for EX and *p* = 0.001 for CON). -IL-10 presented a time x group interaction (*p* = 0.018), and the relative changes were significantly higher in EX when compared with the CON session (*p* < 0.05). -The relative changes in TNF-α tended to be higher in CON when compared with EX immediately post-HD session (*p* = 0.06).
**Esgalhado et al. (2015)** [23]	Non-randomized clinical trial	To assess the effect of acute intradialytic strength physical exercise on oxidative stress and inflammatory responses in HD patients.	16 HD patients (11 women; 44 ± 16.6 years) served as their own controls on a nonphysical-exercise day. Inclusion criteria: maintenance HD ≥6 months; age >18 years and ability to perform strength physical exercise.	Acute (single session) intradialytic physical exercise was performed at 60% of the one-repetition maximum test for 3 sets of 10 repetitions for 4 exercise categories in both lower limbs for 30 min. Blood samples were collected on two different days at exactly the same time (30 min and 60 min after initiating dialysis with and without exercise).	-Antioxidant enzymes activity: superoxide dismutase (SOD), catalase, and glutathione peroxidase (GPx). -Lipid peroxidation marker levels: malondialdehyde (MDA). -Inflammatory marker levels: hs-CRP.	-SOD plasma levels were significantly reduced after acute physical exercise from 244.8 ± 40.7 U/mL to 222.4 ± 28.9 U/mL (*p* < 0.03) and by contrast increased on the day without exercise (218.2 ± 26.5 U/mL to 239.4 ± 38.6 U/mL, *p* < 0.02). -There was no alteration in plasma catalase, GPx, MDA or hs-CRP levels on either day (with or without exercise).
**Dungey et al. (2015)** [31]	Randomized controlled crossover trial	To analyse the immediate effects of a bout of physical exercise during HD on hemodynamic stability, circulating markers of inflammation and aspects of immune function compared to a usual-care HD session.	15 HD patients (9 male; 57.9 ± 10.5 years). All patients completed both study periods: randomization of trial order (exercise trial first, *n* = 7) or (control trial first, *n* = 8). Exclusion criteria: <18 years, had established contraindications to exercise, lower limb vascular access, recent clinically overt infection, prescribed immunosuppressive therapy or an insufficient command of English to consent.	Patients participated in two trial arms during HD treatment usually separated by a week and carried out on the same day of the week. Exercise arm (single session): 5-min warm-up followed by a 30-min bout of intradialytic exercise using a cycle ergometer 60 min into their HD session. (Perceived exertion of “somewhat hard”, 12–14 in RPE). Control arm (single session): patients rested throughout HD.	-Blood pressure: pre-exercise (60 min), immediately post-exercise (100 min), 1 h post-exercise (160 min) and at the end of dialysis (240 min). -Haematology and differential white cell count. -Inflammatory markers: IL-6, TNF-α, IL-1ra and CRP (CRP was measured at 60 min time-point as a reference marker of systemic inflammation). -Cardiac injury markers: heart-type fatty acid-binding protein (h-FABP), myoglobin, cardiac troponin I (cTnI) and creatine kinase MB (CKMB). -Monocyte phenotyping and neutrophil degranulation.	-Blood pressure increased immediately post-exercise; however, 1 h after exercise, blood pressure was lower than resting levels (160 ± 22 vs. 117 ± 25 mmHg). -No differences in h-FABP, cTnI, myoglobin or CKMB were observed between trial arms. -Exercise did not alter circulating concentrations of IL-6 or TNF-α or IL-1ra nor clearly suppress neutrophil function. -CRP concentrations were similar between trial arms (*p* = 0.7).
**Liao et al. (2016)** [37]	Randomized controlled trial	To research whether physical exercise can improve inflammation, the endothelial progenitor cell count (EPC) and bone mineral density (BMD) in HD patients.	40 HD patients were randomly assigned to either an exercise group (*n* = 20, 8 male, 62 ± 8 years) or control group (*n* = 20, 9 male, 63 ± 8 years). Inclusion criteria: maintenance HD ≥6 months; age >18 years and ability to perform strength physical exercise.	Exercise group (EX): 5-min warm-up, 20 min of cycling at the desired workload, and 5-min cool down during 3 HD sessions per week for 3 months. The initiative time was from 30 and 90 min and increased over time according to each patient’s ability until reaching maximal duration. The intensity of exercise was from 12–15 on the RPE. Exercise supervised by an expertise physician and a nurse. Control group (CON): subjects performed a conventional HD session lasting 4 h, without exercise.	-Blood pressure and HR. -Biochemical and anthropometric parameters: creatinine, albumin, calcium, cholesterol, ALT, tHcy, hematocrit, KT/V, nPCR, BW and BMI. -Inflammatory cytokines: IL-6 and hs-CRP. -EPC count: CD133, CD34 and KDR. -BMD, measured using DEXA at L2-L4 and right proximal femur, and iPTH levels measured through radioimmunoassay. -Physical capacity: 6MWT.	After 3 months of exercise: -In EX, increased Albumin (*p* < 0.01), increased BMI (*p* = 0.05) and decreased IL-6 and hs-CRP (*p* < 0.05), which did not occur in CON. -EX exhibited significant increase in circulating CD133, CD34 and KDR-positive EPCs (*p* < 0.05), whereas the number of circulating EPCs did not change in CON. -Bone loss at the femoral neck was attenuated in EX compared with CON (*p* < 0.05). However, there was no significant differences in bone loss of L1-L4 between the 2 groups. -Patients in EX also had a significantly greater 6-MWD after completing the program (*p* < 0.05). -The number of EPCs was correlated with 6-MWD both at the baseline and after 3 months (*p* < 0.0001 and *p* < 0.001, respectively).
**Dungey et al. (2017)** [32]	Non-randomized controlled trial	To determine the impact of a pragmatic 6-month intradialytic exercise program on circulating soluble and cellular markers of chronic systemic inflammation.	Exercise group: 16 HD patients (8 men, 57.0 ± 10.5 years). Non-exercising group: 15 HD patients (10 men, 70.2 ± 13.7 years). Baseline characteristics were compared with 16 healthy age-matched individuals (8 men, 61.5 ± 10.9 years). Inclusion criteria: age >18 years and patients on maintenance HD for at least 3 months.	Exercise program: 6-month intradialytic cycling exercise was performed. Patients exercised 3 times/week, for 30 min each session with a cycle ergometer at rating of “somewhat hard (12–14)” in the RPE. Non-exercising control group: continued HD treatment as per their usual care.	-Inflammatory markers: IL-6, CRP, and TNF-α. -Monocyte phenotypes (classical: CD14^++^CD16^−^, intermediate: CD14^++^CD16^+^ and non-classical: CD14CD16^++^). -Regulatory T cells (Tregs: CD4^+^CD25^+CD127low/−^). -Physical function: STS60 test.	-HD patients were less active than healthy counterparts and had significant elevations in IL-6, CRP, TNF-α, intermediate and non-classical monocytes (all *p* < 0.001). -Physical function (STS60) improved in the exercise group (*p* < 0.001) but not in non-exercise group (*p* = 0.21). -The proportion of intermediate monocytes in the exercising patients was reduced compared with non-exercisers (*p* < 0.01). -Number (but not proportion) of Tregs decreased in the non-exercising patients only (*p* < 0.05). -Training had no significant effect on circulating IL-6, CRP or TNF-α levels.
**Fuhro et al. (2017)** [24]	Randomized controlled crossover trial	To analyse the effects of an acute bout of intradialytic exercise on NK subsets and circulating biomarkers in patients with ESRD.	9 HD patients (2 men, 64.88 ± 1.98 years). All participants were randomly assigned to perform 2 HD sessions with different protocol: exercise trial or control trial. Inclusion criteria: patients aged between 45 and 70 years, performed HD 3 times/week for at least 6 months prior to the study, urea reduction ratio (URR) ≥65%, not practicing any physical activity.	All trials were performed with an interval of at least 1 week (7 days). Exercise trial: single bout of 20-min intradialytic exercise in the second hour of HD session, using lower a limb cycle ergometer. (Perceived exertion of 6–7 in the MBS). After the trial, patients performed stretches of lower limb muscles. Control trial: a conventional rested HD session within exercise.	-NK cells and their subsets (CD3-CD56^bright^ and CD3-CD56^dim)^. -Systemic cortisol concentrations. -CRP. -Creatine kinase activity. -Urea and creatinine. -Anthropometric measures: body mass, weight and BMI. -Physical capacity: 6MWT. Peripheral blood sample was collected at baseline, during HD and immediately after HD in each trial.	-HD therapy induced a significant decrease in NK cell frequency (*p* = 0.039), CD3-CD56^bright^ (*p* = 0.04) and CD3-CD56^dim^ (*p* = 0.036). -No significant alterations were observed in NK and NK subsets during and after intradialytic exercise trial (*p* > 0.05). -Neither trial altered CRP levels or serum CK activity during and after HD therapy (*p* > 0.05). -HD therapy increased cortisol concentrations after HD therapy (*p* = 0.034).
**Wong et al. (2017)** [33]	Randomized controlled crossover trial	1. To determine the effect of intradialytic exercise on blood endotoxin levels and markers of inflammation. 2. To determine if endotoxin levels were falsely elevated in HD patients due to (1→3)-β-D glucan-based activation of the LAL assay.	10 HD patients who regularly performed intradialytic exercise (70% male, mean age 62 years). Half of the studies were randomly assigned to be carried with the non-exercise session following the exercise session. The remaining studies were carried out with the sessions completed in reverse order.	Patients were studied on 2 separate HD sessions. First session: patients were asked to abstain from intradialytic exercise during HD (non-exercise day). Second session: patients were asked to perform their routine intradialytic exercise program with a cycle ergometer (exercise day). 2-min warm-up, 30-min cycling. Perceived Exertion was recorded with the RPE. These sessions were carried out 1 week apart.	-Inflammation markers: IL-6, hs-CRP and TNF-α. -Blood endotoxin levels by LAL assay (limulus amebocyte lysate). -(1→3)-β-D glucan (BG) by Fungitell assay. -D-lactate (a sensitive marker of intestinal ischaemia). Blood samples were collected at 3 time-points: pre-dialysis, immediately post-dialysis and 1–3 h post-dialysis.	-Patients exercised for a mean of 100 min (95% CI 57.7-142.2) and the mean of RPE was 12 (“fairly light”). -With the exception of one sample, all samples tested negative for endotoxin. -Intradialytic exercise attenuated the rise of IL-6, TNF-α and hs-CRP after the HD procedure. -No significant changes in (1→3)-β-D glucan (BG) or D-lactate were observed in either group after the intervention.
**Gonçalves da Cruz et al. (2018)** [25]	Randomized controlled trial	To assess the effect of 12 weeks of intradialytic aerobic training on serum levels of IL-1β, IL-6, IL-8, IL-10, TNF-α and functional capacity.	30 sedentary volunteers in HD treatment were randomly assigned to either exercise (15 subjects, 10 men, aged 43.5 ± 14.4 years) or control group (15 subjects, 3 men, aged 39.9 ± 13.5 years). Inclusion criteria: aged 18–65 years, in HD treatment for at least 3 months, sedentary and healthy conditions for practice exercise.	Exercise group (EX): were summited to 12 weeks of aerobic training performed on a cycle ergometer for 30 min at an intensity of 6–7 in the RPE, 3 times a week. Control group (CON): kept the daily habits.	-Anthropometric measures: body mass, weight and BMI. -Kt/V Index. -Inflammatory markers: IL-1β, IL-6, IL-8, IL-10 and TNF-α. -Functional capacity: 6MWT.	-After 12 weeks, only EX presented a significant reduction in serum levels of IL-1β, IL-6, IL-8, TNF-α (*p* < 0.05 in all of them) and an increase in serum levels of IL-10 and 6MWT (*p* < 0.05 in both). -There was also a significant reduction in BMI in EX (*p* < 0.05), which was not observed in CON.
**Figueiredo et al. (2018)** [26]	Randomized controlled trial	To research and compare the isolated and mixed effects of both inspiratory muscle training and aerobic training on respiratory and functional measures, inflammatory biomarkers, redox status and health-related quality of life in HD patients.	37 HD patients were randomized into 3 groups: Inspiratory Muscle training (IMT): *n* = 11; mean age = 52.8 years (43.1–62.5). Aerobic Training (AT): *n* = 13; mean age = 49.5 years (41.6–57.3). Combined Training (CT): *n* = 13; mean age = 45.2 years (34.8–55.5). Inclusion criteria: HD patients >18 years, clinically stable and in response to exercise.	8 weeks intradialytic exercise program (3 times/week). IMT: 3 sets of 15 deep inspirations at the equipment mouthpiece and rested for 60 s with the linear loud adjusted to 50% MIP. AT: 5-min warm-up, 30 min by cycle ergometer and 5-min cooling-down. Loud was adjusted between 3 and 5 in the RPE. Speed remained ≥50 rpm. CT: IMT was performed immediately before AT. Prior to the interventions, all volunteers underwent an 8-week control period (no training).	-Inflammatory parameters: IL-6, sTNFR1 and sTNFR2, adiponectin, resistin and leptin. -Redox status: SOD, catalase, TBARS and FRAP plasma levels. -HRQoL: KDQOL-SF. -Functional capacity: incremental shuttle walk test. -MIP. -Lower limb strength: sit-to-stand test of 30 s. -Anthropometric parameters: weight, BMI, WC and body fat percentage. -Exercise adherence. -Measures were taken at T_0_, T_8_ and T_16_ (baseline, 8 and 16 weeks).	-increased MIP, functional capacity, lower limbs strength and resistin levels, and decreased sTNFR2 levels at T_16_, compared to T_0_ and T_8_, in all groups (*p* < 0.001), without between-group differences. -increased adiponectin levels (*p* < 0.001) and fatigue domain of HRQoL (*p* < 0.05) at T_16_ only in CT. -No significant differences in IL-6, sTNFR1 and leptin levels, nor in any of the oxidative stress parameters. -Exercise adherence was >94.9% in the 3 groups.
**Martin et al. (2018)** [34]	Non-randomized crossover trial	To evaluate the acute effect of intradialytic exercise (IDE) on microparticle (MP) number and phenotype, and their ability to induce endothelial cell reactive oxygen species (ROS) in vitro.	11 patients (7 males, mean age = 59 ± 10 years) were studied during a routine HD session and one where they exercised. Inclusion criteria: HD patients > 18 years, clinically stable and in response to exercise.	Patients participated in 2 trial arms, separated by a week and carried out on the same day each week. Exercise arm (EX): 60 min into their HD treatment, patients performed a 5-min warm-up followed by a 30-min bout of IDE using a cycle ergometer.	-Total number of MPs over the course of HD in exercising and non-exercising CKD patients. -Procoagulant MPs over the course of HD in exercising and non-exercising CKD patients. -Effects of HD and IDE on the cellular origin of MPs (platelets, EC, neutrophils and monocytes) -ROS-inducing effects of MPs from dialyzing and IDE. Measures were taken at 60 min of HD (pre-EX), 100 min into HD (post-EX), 160 min into HD (60 min post-EX) and at the end of HD.	MP number increased during HD (*p* < 0.001) as did phosphatidylserine+ (*p* < 0.05), platelet-derived (*p* < 0.01) and percentage procoagulant neutrophil-derived MPs (*p* < 0.05), but this was not affected by IDE. However, MPs collected immediately and 60 min after IDE (but not later) induced greater ROS generation from cultured endothelial cells (*p* < 0.05), suggesting a transient proinflammatory event.
**Dong et al. (2019)** [38]	Randomized controlled trial	To research the effect of intradialytic resistance exercise on inflammation markers and sarcopenia indices in maintenance hemodialysis (MHD) patients with sarcopenia.	41 MHD patients with sarcopenia were randomized into an exercise group (*n* = 21; 9 male, mean age 59.0 [32.5, 66.5]) and a control group (*n* = 20; 12 male, mean age 62.5 [50.5, 70.0]). Inclusion criteria: aged 18–80 years, stable dialysis time ≥3 months, no central systemic disease, can walk independently, no physical disability and muscle strength ≥III.	Exercise group (E): patients received progressive intradialytic resistance exercise with high or moderate intensity (>15 in the RPE) for 12 weeks at 3 times/week (using their own body weight and elastic balls). 5-min warm-up followed by 1–2 h bout of intradialytic exercise. Control group (C): patients only received routine HD care.	-Inflammatory parameters: IL-6, IL-10, TNF-α and hs-CRP. -Physical activity status: maximum grip strength, daily pace, and physical activity level (questionnaire). -Dialysis adequacy: Kt/V. -Body component-related nutritional indicators: BIA, SMI, SMM, FM, FMI, FFM, FFMI and WHR. The volunteers were evaluated at baseline, and after 12 weeks (intervention period).	-After 12 weeks, a significant difference in physical activity status, Kt/V, and hs-CRP was found between groups E and C (*p* < 0.05). -IL-6 and IL-10, as representatives of anti-inflammatory factors, did not significantly change after the exercise intervention (*p* > 0.05). -The pro-inflammatory factor TNF-α decreased significantly after the exercise intervention (*p* < 0.05).
**Suhardjono et al. (2019)** [39]	Randomized controlled trial	To determine the role of intradialytic exercise performed 2 times per week on physical capacity, inflammation and nutritional status in HD patients and to determine which exercises were more suitable for this population.	120 HD participants were randomized into 3 groups: Aerobic group: *n* = 42; 28 men, mean age 49.78±11.65 years. Aerobic and resistance group: *n* = 39; 21 men, mean age 46.38 ± 14.19 years. Control group: *n* = 39; 18 men, mean age 50.54 ± 10.83 years. Inclusion criteria: HD patients > 18 years, maintenance HD for >3 months.	A physical exercise program was carried out twice a week for 12 weeks. Aerobic exercise program: 30 min using a cycle ergometer. Resistance exercise program: 3 sets of 10 repetitions of ankle weightlifting with 1-min rest between each repetition. (Exercise intensity: 12–13 in the RPE).	-Physical capacity: skeletal muscle mass index (by bioimpedance), muscle strength (by dynamometer) and gait speed and physical component of KDQOL. -Nutritional status: malnutrition inflammation score (MIS). -Inflammation: hs-CRP. The volunteers were evaluated at baseline, and after 12 weeks.	-A significant increase in lower extremity strength occurred in the aerobic and combined exercise groups compared to the lower extremity strength of the control group. -A significant increase in the physical component score (PCS) of the KDQL-SF^TM^ instrument was also present in the aerobic training and combined exercise groups compared to the PCS of the control group. -There were no significant differences between the two exercise groups in any outcome. -There was no significant effect on handgrip, gait speed, inflammation markers, or nutritional status.
**Torres et al. (2020)** [40]	Uncontrolled trial	To evaluate the effects of exercise training during HD sessions on physical functioning, body composition and nutritional and inflammatory status.	36 patients on HD therapy (61% male, mean age 56 ± 17 years). Inclusion criteria: age >18 years, clinical stability with no hospitalization in the previous 3 months and 3-month prevalence in HD program before inclusion.	3-month exercise training was performed, 3 weekly sessions from 45 to 50 min. The exercise routine was performed during the first hour of each HD session and was based on sets of bending and stretching of arms and legs repetitions, hip abduction and hand grip exercises. The objective was to perform 4 sets of 3 exercises in the lower limbs and 4 sets of 5 exercises in the upper limb. The exercise program was monitored by the staff of HD units in each HD session and the weights lifted and hand grip resistances were progressively increased according to the progression of the patients.	-Functional ability: 6MWT, STS-30 and upper extremity strength measured by dynamometry. -Body composition: bioimpedance, weight, BMI, lean tissue index and fat tissue index. -Laboratory parameters: cholesterol, LDL, HDL, triglycerides, proteins, albumin, hemoglobin and ferritin. -Inflammatory parameters: CRP. The volunteers were evaluated at baseline, and after 3 months (intervention period).	After 3 months of exercise training: -Functional ability (6MWT, STS-30 and dynamometry) improved significantly (*p* < 0.001, *p* = 0.003 and *p* < 0.001, respectively). -Body composition improved with an increase of BMI (*p* = 0.01) at the expense of lean tissue index (*p* = 0.038) and lipid parameters with LDL-cholesterol decrease (*p* = 0.03) and lower serum triglyceride levels (*p* = 0.006). -In addition, a decrease in iron (*p* = 0.029) and erythropoietin (*p* = 0.023) requirements was observed. -There were no significant changes in albumin, total proteins or CRP levels.
**Oliveira e Silva et al. (2019)** [27]	Randomized controlled trial	To assess the impact of aerobic training on non-traditional cardiovascular risk factors in CKD patients on HD.	30 HD patients were randomized into 2 groups: Intervention group (IG): *n* = 15; 7 males, mean age 50 ± 17.2 years. Control group (CG): *n* = 15; 8 males, mean age 58 ± 15.0 years. Inclusion criteria: HD patients > 18 years, maintenance HD for >3 months, on stable medication, and who did not present contraindications for physical exercise.	IG: 4-month intradialytic aerobic physical training performed during HD session 3 times a week, during the first 2 hours of the session, with a cyclo-ergometer. The aerobic training lasted 30 min without interruption, at between 65 and 75% HR_max_, with a Borg scale score around 13. CG: no intervention.	-Physical activity: short version of the International physical Activity Questionnaire (IPAQ), VO_2_ max and HR_max_. -Left ventricular mass (LVM) and left ventricular hypertrophy (LVH). -Aldosterone concentration and flow-mediated vasodilatation (FMV). -Arterial stiffness. -Inflammatory measures: CRP. The volunteers were evaluated at baseline, and after 4 months.	- In IG, there was a statistically significant improvement in FMV (*p* = 0.002) and a reduction in LVH (*p* = 0.006) and serum aldosterone (*p* = 0.016). -There was no statistically significant difference regarding alterations of VO_2_ max and arterial stiffness between groups or moments. -There was an increase in CRP in CG (*p* = 0.002) after 4 months.
**Lopes et al. (2019)** [28]	Randomized controlled trial	To compare the effects of high vs moderate loads of intradialytic resistance training on body composition, sarcopenia prevalence, functional capacity, inflammatory markers and quality of life in individuals on HD.	80 HD patients were randomized into 3 groups, 2 intervention groups and 1 control group. Finally, 50 subjects were analyzed. **Intervention groups:** High-load intradialytic group (HLG): *n* = 14; mean age 48.1 ± 10.8 years. Moderate-load intradialytic group (MLG): *n* = 16; mean age 56.2 ± 12.5 years. Patients with adherence <70% were excluded. **Control group (CG):** *n* = 20; mean age 56.9±12.4 years.	12-week intradialytic resistance training (RT) was performed 3 times a week; each session involved 5 exercises: unilateral knee extension, knee curl, hip flexion, seated calf raises and leg press. Individuals were asked to perform as many repetitions as possible. HLG: 8–10 repetitions. MLG: 16–18 repetitions. CG: stretching exercises. The subject perception exertion was recorded using the OMNI Resistance Exercise Scale.	-Body composition. -Sarcopenia prevalence. -Skeletal muscle index (SMI). -Functional capacity: Short Physical Performance Battery and Timed Up and Go test. -Inflammatory markers: IL-6, IL-10 and TNF-α. -Quality of life (KDQOL). -Adherence to the resistance training program. The volunteers were evaluated at baseline, and after 12 weeks.	-High-load intradialytic RT was associated with gains in lean leg mass (*p* = 0.04). -SMI was improved in both RT groups when compared with CG (*p* = 0.01). -Regarding QoL, only HLG showed a decreased pain perception (*p* = 0.04) and an increase in physical function (*p* = 0.04) compared to CG. -No differences in IL-6, IL-10, and TNF-α concentrations were observed after intervention. -3 subjects from HLG and 3 from MLG were excluded for not performing 70% of the sessions.
**Sovatzidis et al. (2020)** [42]	Randomized controlled trial	To investigate the effectiveness on redox status of a 6-month intradialytic exercise training program, inflammation and physical performance in ESRD.	20 HD patients were randomly assigned to either an intradialytic training group or a control group for 6 months. Intradialytic training group (TR): *n* = 10; mean age 52.8 ± 17.1 years. Control group (CG): *n* = 10; mean age 53 ± 7.6 years. Inclusion criteria: maintenance HD for ≥12 months, no use of antioxidant supplements and ability to execute a stationary bike workout.	6-month intradialytic cardiovascular exercise program with cycle ergometer was performed, 3 times a week. Each exercise session consisted of 5-min warm-up, cycling at the desired workload for a self-selected time (depending on each participant’s tolerance) and 5-min cool-down. Exercise intensity: 11–13 in the RPE. CG: patients only received routine HD care.	-Anthropometric profile: body mass and height, BMI, body composition. -Physical performance: VO_2_ peak. -Functional capacity: NSRI walk test and STS-60. -Quality of life: short form-36 (SF36). -Redox status: TBARS, PC, GSH, GSSG, GSH/GSSG, TAC and CAT. -Inflammatory markers: hs-CRP.	-VO_2_ peak increased by 15% only in TR (*p* < 0.01). -Performance in NSRI, STS-60 and SF-36 improved by 4–13% only in TR (*p* < 0.01). -Exercise training reduced TBARS (by 28%), PC (by 31%) and hs-CRP (by 15%), and elevated GSH (by 52%), GSH/GSSG (by 51%), TAC (by 59%) and CAT (by 15%) (*p* < 0.01).
**Corrêa et al. (2020)** [29]	Randomized controlled trial	To assess the effects of 3 months of resistance training (RT) on sleep quality, redox balance, nitric oxide (NO) bioavailability, inflammation profile and asymmetric dimethylarginine (ADMA) in patients undergoing HD.	55 men undergoing maintenance HD were randomized into either a control or resistance training group. Resistance training group (RTG): *n* = 30; mean age 66.0 ± 4.0 years. Control group (CG): *n* = 25; mean age 65.7 ± 3.8 years. Inclusion criteria: HD patients ≥ 50 years, maintenance HD for at least 3 months.	The patients on RTG were enrolled in a structured periodized program of 50-minute sessions, 3 sessions per week for 12 weeks undergoing HD (intradialytic exercise). RT repetitions balanced concentric and eccentric lifting phases with TheraBand (each phase lasted 2 seconds), verified and supervised by a strength and conditioning specialist. RTG sessions consisted of 11 strength exercises where the upper and lower limb muscles were worked with weights and Thera-bands.	-Biochemical parameters. -Sleep quality. -Redox profile: TBARS, and total antioxidant capacity (Trolox equivalent). -Inflammatory profile: TNF-α and IL-10. -Biomarkers of endothelial function: NO and ADMA. -Muscle strength. The volunteers were evaluated at baseline, and after 12 weeks (intervention period).	-Total sleep time and sleep efficiency improved in RTG as compared with pre-training and CG (*p* < 0.05). -TBARS and TNF-α decreased, while total antioxidant capacity (Trolox equivalent) and IL-10 increased in RTG in the post-training as compared with pre-training and CG (*p* < 0.0001). -The CG participants also experienced a decrease in Trolox (*p* < 0.0001). -NO_2_^−^ increased and ADMA decreased in RT when compared to pre-training and CG (*p* < 0.0001). -ADMA increased in CG over time (*p* < 0.0001).
**Meléndez et al. (2022)** [41]	Randomized controlled trial	To evaluate the effect of a 4-month combined strength and aerobic endurance exercise program on biomarkers of inflammation and oxidative stress in patients with CKD in HD.	71 HD patients were randomized in 2 groups who performed aerobic and strength exercise combined. Intradialysis exercise group: *n* = 36; mean age 70.55 ± 13.26 years. Exercise group Domicile: 35; mean age 67.26 ± 16.21 years.	4 months, 3 sessions per week, 12–15 RPE, until reaching 60 min per session. Aerobic exercise with cycloergometer and strength exercises with Thera-bands and weights.	-Inflammatory parameters: IL-6, TNF-α, CRP, MCP-1, ICAM-1. -Oxidative stress parameters: MDA, PC, GSH, GSSG, GSH/GSSG.	IL-6 plasma levels showed a significant decrease in the intra-dialysis group after exercise (*p* = 0.03), while CRP levels decreased significantly in the home-based group (*p* = 0.03). MCP-1, TNF-α, ICAM-1 and the oxidative stress markers MDA, GSH and GSSG, did not undergo significant changes after the intervention.
**March et al. (2022)** [35]	Randomized controlled trial	To investigate the effect of aerobic exercise on circulating endotoxins and cytokines in patients receiving haemodialysis.	92 HD patients (mainly males) were randomized into 2 groups: aerobic exercise (*n* = 46; mean age 61 ± 14 years) and control group (*n* = 46; mean age 53 ± 15 years). Inclusion criteria: individuals receiving HD; age >18 years.	6 months intradialytic exercise program (3 times/week). Aerobic group: 30 minutes at 12–14 RPE. Control group: Usual care.	-Endotoxin measurement -Cytokine analysis (IL-6, IL-10, TNF-α, CRP).	Circulating levels of endotoxins did not change from baseline to 6 months in the aerobic group, while there was a small increase in control group. No differences between groups at 6 months (*p* = 0.137). No significant differences between groups after 6 months in any cytokine.
**Highton et al. (2022)** [36]	Randomized controlled trial	To investigate how regular, moderate-intensity exercise affects inflammation in haemodialysis patients	40 HD patients (mainly males) were randomized into 2 groups: aerobic exercise (*n* = 20; mean age 56.8 ± 14 years) and control group (*n* = 20; mean age 51.4 ± 18.1 years). Inclusion criteria: Individuals >3 months HD; age >18 years	6 months intradialytic exercise program (3 times/week). Aerobic group: 5 minutes of warm-up, 30 minutes of exercise and 5 minutes of cool-down from 12 to14 RPE. Control group: Usual care.	-Cytokines (IL-2, IL-6, IL-10, IL-17a, TNF-α) -Chemokines (IL-8, MCP-1).	No differences between groups in IL-6 and IL-10 values (*p* > 0.060). No differences between groups in IL-17a values (*p* > 0.262). Strong evidence to suggest a significant time*group interaction in TNF-α (*p* = 0.001), but no further statistical differences upon post hoc analysis. No differences in IL-8 and MCP-1 at baseline (*p* > 0.101) and after treatment (*p* > 0.151).

AC: Abdominal circumference; ADMA: Asymmetric dimethylarginine; ALT: alanine aminotransferase; AMA: Arm muscle area; AT: Aerobic training; BG: (1–3)-β-D glucan; BIA: Bioimpedance analysis; BMI: Body Mass Index; BW: body weight; CA-IMT: Carotid intima-media thickness; CAT: Catalase activity; CI: Confidence interval; CK: Creatinine kinase; CKD: Chronic Kidney Disease; CKMB: Creatine kinase MB; CON: Control; CT: Combined training; cTnI: Cardiac troponin I; DEXA: Dual-energy X-ray absorptiometry; EC: Endothelial cells; eGFR: Estimated glomerular filtration rate (mL/min per 1.73 m^2^); ESRD: End-stage renal disease; EPC: Endothelial progenitor cell; EX: Exercise; FM: Fat mass; FMI: Fat mass index; FFM: Fat-free body weight; FFMI: Fat-free body mass; FRAP: Ferric reducing antioxidant power; GPx: Glutathione peroxidase; GSH: reduced glutathione; GSSG: oxidized glutathione; HD: Hemodialysis; HDL: High density lipoprotein; h-FABP: Heart-type fatty acid binding protein; HLA-DR: Human leucocyte antigen; HRQoL: Health-related quality of life; HR: Heart rate; hs-CRP: C-Reactive Protein, High Sensitivity; HRmax: Maximal heart rate; hs-CRP: high-sensitivity C-reactive protein; IL-6: Interleukin 6; IL-10: Interleukin 10; IL-17a: Interleukin 17a; INF-γ: Interferon-gamma; iPTH: intact parathyroid hormone; IPAQ: International physical Activity Questionnaire; KDQOL-SF: Kidney Disease Quality of Life—Short Form; LDL: Low-density lipoprotein; MET: Metabolic equivalent of task; MAFbx: Muscle atrophy F-box; MPC-1: Monocyte chemoattractant protein 1; MHD: Maintenance hemodialysis; MBS: Modified Borg Scale; MCP-1: Monocyte chemoattractant protein-1; MDA: Malondial-dehyde; MIP: Maximal Inspiratory Pressure; MIS: Malnutrition-inflammatory score; MP: Microparticles; MWD: Minute walk distance; MyoD: Myogenic Differentiation 1; nPCR: normalized protein catabolic rate; NSRI: North Staffordshire Royal Infirmary walk test; OMNI-RES: OMNI resistance exercise scale; OR: Odds ratio; PAEE: Physical activity energy expenditure; P-Akt: Phosphorylated Akt; PC: Protein carbonyl; P-eEf2: Phosphorylation of eukaryotic elongation factor 2; Pre-EX: Before exercise; Post-EX: After exercise; PEW: Protein-Energy Wasting; RCT: Randomized controlled trial; REE: Resting Energy Expenditure; RETP: Resistance Exercise Training Program; 1RM: 1-Repetition maximum test; SMI: Skeletal muscle index; RPE: Borg Rating of Perceived Exertion Scale; RE: Resistance exercise; RS-2: High amylose maize resistant starch type 2; SBP: Systolic blood pressure; SGA: Subjective global assessment; SMI: Skeletal muscle mass index; SMM: Skeletal muscle mass; SOD: Superoxide dismutase; STS 60:60 second sit-to-stand test; STS 30:30 second sit to stand test; SWA: Sense Wear Pro2 Armband, Body-Media Inc, Pittsburgh, PA; T_0_: At baseline; T_8_: At 8 weeks (after control period), T_16_: At 16 weeks (after interventions); T_3_: At 3 months; tHcy: total homocysteine; TAC: Total antioxidant capacity; TBARS: Thiobarbituric acid reactive substances; TG: Triglycerides; TNF-α: Tumoral necrosis factor-alpha; VO_2peak_: Peak O2 uptake; WC: Waist Circumference; W_peak_: Peak workload; WHR: Waist-to-hip ratio.

**Table 2:** Methodological quality evaluation of the clinical trials using the PEDro Scale for assessing the risk of bias in randomized and non-randomized trials.

Scale “Physiotherapy Evidence Database (PEDro)” to Analyze the Methodological Quality of Clinical Studies												
Authors	Specified Selection Criteria	Randomization	Hidden Assignment	Similar Groups to Start	Blinded Subjects	Blinded Therapists	Blinded Raters	Outcomes 85%	Treatment or Intention to Treat	Comparison between Groups	Point Measures Variability	Outcome
**Afshar et al. (2010)** [19]	Yes	Yes	Yes	Yes	No	No	Yes	Yes	Yes	Yes	Yes	**9**
**Moraes et al. (2012)** [20]	No	No	No	No	No	No	Yes	Yes	Yes	No	Yes	**4**
**Golebiowski et al. (2012)** [30]	Yes	No	No	No	No	No	Yes	Yes	Yes	No	Yes	**5**
**Moraes et al. (2014)** [21]	Yes	No	No	Yes	No	No	Yes	No	Yes	No	Yes	**5**
**Peres et al. (2015)** [22]	Yes	Yes	Yes	Yes	No	No	Yes	Yes	Yes	Yes	Yes	**9**
**Esgalhado et al. (2015)** [23]	Yes	No	No	No	No	No	Yes	Yes	Yes	Yes	Yes	**6**
**Dungey et al. (2015)** [31]	Yes	Yes	Yes	Yes	No	No	Yes	Yes	Yes	Yes	Yes	**9**
**Liao et al. (2016)** [37]	Yes	Yes	Yes	Yes	No	No	Yes	Yes	Yes	Yes	Yes	**9**
**Dungey et al. (2017)** [32]	Yes	No	No	No	No	No	Yes	No	Yes	Yes	Yes	**5**
**Fuhro et al. (2017)** [24]	Yes	Yes	Yes	Yes	No	No	Yes	Yes	Yes	Yes	Yes	**9**
**Wong et al. (2017)** [33]	Yes	Yes	Yes	Yes	No	No	Yes	Yes	Yes	Yes	Yes	**9**
**Gonçalves da Cruz et al. (2018)** [25]	Yes	Yes	Yes	Yes	No	No	Yes	Yes	Yes	Yes	Yes	**9**
**Figueiredo et al.** [26] **(2018)**	Yes	Yes	Yes	Yes	No	No	Yes	Yes	Yes	Yes	Yes	**9**
**Martin et al. (2018)** [34]	Yes	Yes	Yes	Yes	No	No	Yes	Yes	Yes	Yes	Yes	**9**
**Dong et al. (2019)** [38]	Yes	Yes	Yes	Yes	No	No	Yes	Yes	Yes	Yes	Yes	**9**
**Suhardjono et al. (2019)** [39]	Yes	Yes	Yes	Yes	No	No	Yes	Yes	Yes	Yes	Yes	**9**
**Torres et al. (2020)** [40]	Yes	No	No	No	No	No	Yes	Yes	Yes	No	Yes	**5**
**Oliveira e Silva et al. (2019)** [27]	Yes	Yes	Yes	Yes	No	No	Yes	Yes	Yes	Yes	Yes	**9**
**Lopes et al. (2019)** [28]	Yes	Yes	Yes	Yes	No	Yes	Yes	Yes	Yes	Yes	Yes	**10**
**Sovatzidis et al. (2020)** [42]	Yes	Yes	Yes	Yes	No	No	Yes	Yes	Yes	Yes	Yes	**9**
**Corrêa et al. (2020)** [29]	Yes	Yes	Yes	Yes	No	No	Yes	No	Yes	Yes	Yes	**8**
**Meléndez et al. (2022)** [41]	Yes	Yes	Yes	Yes	No	Yes	Yes	Yes	Yes	Yes	Yes	**10**
**March et al. (2022)** [35]	Yes	Yes	Yes	Yes	No	Yes	Yes	Yes	Yes	Yes	Yes	**10**
**Highton et al. (2022)** [36]	Yes	Yes	Yes	Yes	No	Yes	Yes	Yes	Yes	Yes	Yes	**10**

Result on the PEDro scale: 9–10 (excellent), 6–8 (good), 4–5 (acceptable) and <4 (poor).

**Table 3:** Methodological quality evaluation of the clinical trials using the Cochrane Risk of Bias Tool for assessing the risk of bias in randomized trials.

Risk of Bias of Cochrane Collaboration Tool of Randomized Controlled Trials Included							
Author (Year)	Random Sequence Generation	Allocation Concealment	Blinding (Participants and Personnel)	Blinding (Outcome Assessment)	Incomplete Outcome Data	Selective Reporting	Other Sources of Bias
**Afshar et al. (2010)** [19]	Low risk	High risk	High risk	Unclear	Low risk	Low risk	Low risk
**Peres et al. (2015)** [22]	Low risk	High risk	High risk	Unclear	High risk	Low risk	Low risk
**Dungey et al. (2015)** [31]	Low risk	Low risk	High risk	Low risk	Low risk	Low risk	Low risk
**Liao et al. (2016)** [37]	Low risk	Low risk	High risk	Low risk	Low risk	Low risk	Low risk
**Fuhro et al. (2017)** [24]	Low risk	High risk	High risk	Unclear	Low risk	Low risk	Low risk
**Wong et al. (2017)** [33]	Low risk	High risk	High risk	Unclear	Low risk	Low risk	Low risk
**Gonçalves da Cruz et al. (2018)** [25]	Low risk	Low risk	High risk	Unclear	Low risk	Low risk	Low risk
**Figueiredo et al. (2018)** [26]	Low risk	Low risk	High risk	Low risk	Low risk	Low risk	Low risk
**Dong et al. (2019)** [38]	Low risk	High risk	High risk	Unclear	Low risk	Low risk	Low risk
**Suhardjono et al. (2019)** [39]	Low risk	Low risk	High risk	Low risk	Low risk	Low risk	Low risk
**Oliveira e Silva et al. (2019)** [27]	Low risk	High risk	High risk	Low risk	Low risk	Low risk	Low risk
**Lopes et al. (2019)** [28]	Low risk	Low risk	High risk	Low risk	Low risk	Low risk	Low risk
**Sovatzidis et al. (2020)** [42]	Low risk	Unclear	High risk	Low risk	Low risk	Low risk	Low risk
**Corrêa et al. (2020)** [29]	Low risk	High risk	High risk	Unclear	High risk	Low risk	Low risk
**Meléndez et al. (2022)** [41]	Low risk	Unclear	High risk	Low risk	Low risk	Low risk	Low risk
**March et al. (2022)** [35]	Low risk	Unclear	High risk	Low risk	Low risk	Low risk	Low risk
**Highton et al. (2022)** [36]	Low risk	Unclear	High risk	Low risk	Low risk	Low risk	Low risk

**Table 4:** Methodological index for non-randomized studies (MINORS) to assess the methodological quality and risk of bias of the included observational studies. Items are scored 0 (not reported), 1 (reported but inadequate) or 2 (reported and adequate), with the global ideal score being 16 for non-comparative studies and 24 for comparative studies.

Methodological Index for Non-Randomized Studies (MINORS)													
Authors	A Clearly Stated Aim	Inclusion of Consecutive Patients	Prospective Collection of Data	Endpoints Appropriate To The Aim Of The Study	Unbiased Assessment of the Study Endpoint	Follow-Up Period Appropriate to the Aim of the Study	Loss to Follow Up Less than 5%	Prospective Calculation of the Study Size	An Adequate Control Group *	Contemporary Groups *	Baseline Equivalence of Groups *	Adequate Statistical Analyses *	Outcome
**Moraes et al. (2012)** [20]	2	0	1	2	1	2	0	1	0	0	0	2	**11**
**Golebiowski et al. (2012)** [30]	2	2	2	2	1	2	0	2	0	0	0	2	**15**
**Moraes et al. (2014)** [21]	2	2	2	2	1	2	0	2	0	0	0	2	**15**
**Esgalhado et al. (2015)** [23]	2	2	2	2	1	2	2	1	1	0	2	1	**18**
**Dungey et al. (2017)** [32]	2	2	2	2	1	2	0	1	2	2	1	2	**19**
**Martin et al. (2018)** [34]	2	2	2	2	1	2	2	0	1	0	1	2	**17**
**Torres et al. (2019)** [40]	2	2	2	2	0	2	0	2	0	0	0	2	**14**

The items are scored 0 (not reported), 1 (reported but inadequate) or 2 (reported and adequate). The global ideal score being 16 for non-comparative studies and 24 for comparative studies. * Additional criteria in case of comparative study.

**Table 5:** Summary of findings for clinical trials, including the GRADE quality of evidence assessment.

Quality Assessment of Aerobic Exercise Studies Improving Systemic Inflammation Biomarkers							
Number of Studies (Subjects)	Risk of Bias	Inconsistency	Indirectness	Imprecision	Publication Bias	Quality	Grade of Recommendation
14 (*n* = 388)	Serious *	Serious ^‡^	Not serious	Not serious	Not serious	Low quality	Weak in favor
**Quality Assessment of Resistance Exercise Studies Improving Systemic Inflammation Biomarkers**							
**Number of Studies (Subjects)**	**Risk of Bias**	**Inconsistency**	**Indirectness**	**Imprecision**	**Publication Bias**	**Quality**	**Grade of Recommendation**
7 (*n* = 316)	Serious *	Serious ^‡^	Not serious	Not serious	Not serious	Low quality	Weak in favor

* Blinding and/or allocation concealment issues. ^‡^ Point estimates varied among studies. The GRADE system establishes 4 degrees of evidence (high, moderate, low and very low), and 2 degrees of recommendation (strong or weak) for or against the intervention; For each item a judgment is made (very serious, serious, not serious).

## Data Availability

The data presented in this study are available upon request from the corresponding authors.
